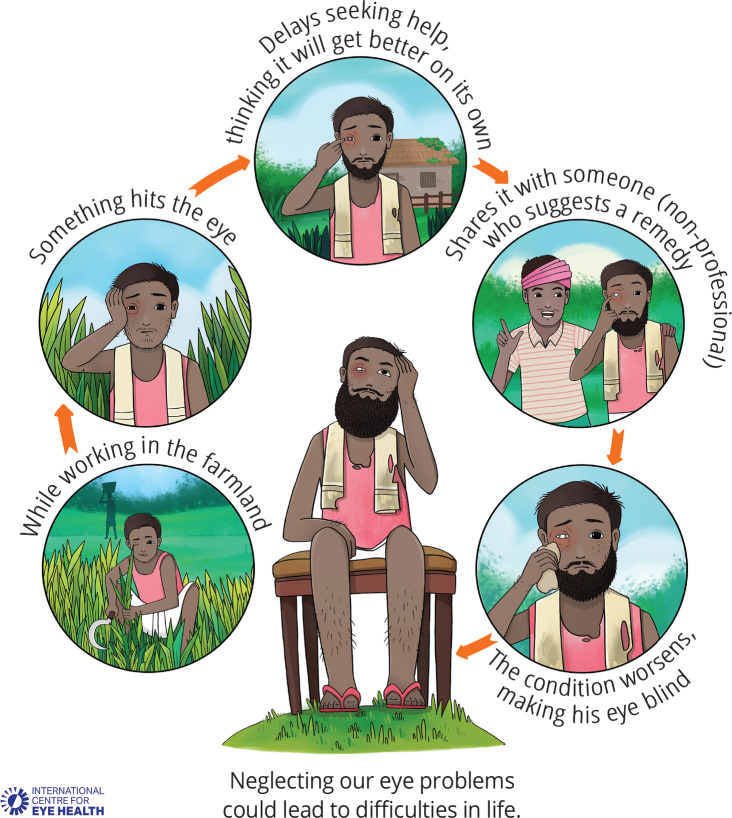# A Tale of Two Farmers

**Published:** 2025-01-31

## Sukhiya: a happy outcome

*If you get injured and the eye gets red,*

*Make sure you stop and use your head,*

*Get to the nearest health centre quick,*

*And stop yourself from getting sick.*

**Figure:**
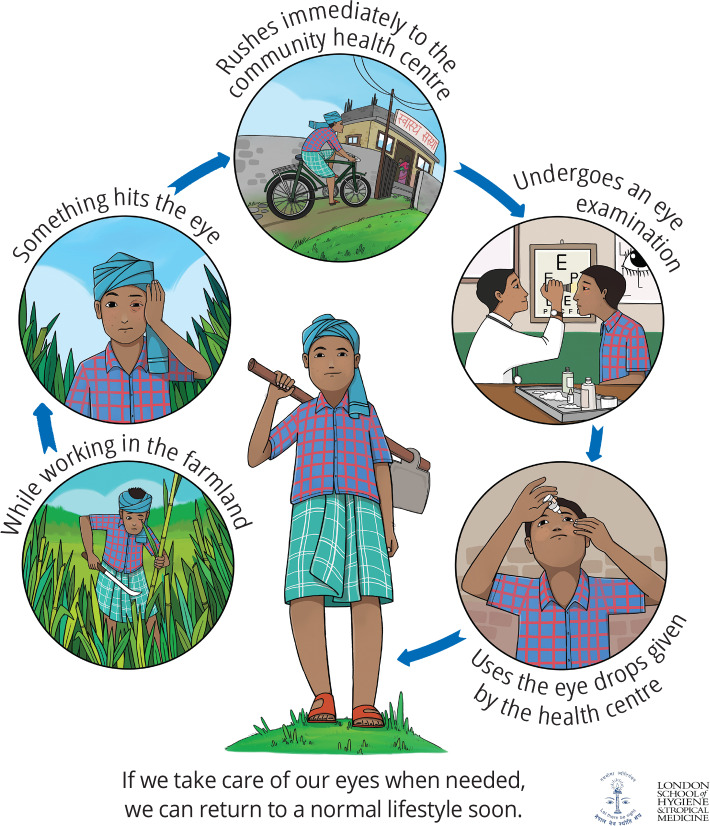
ILLUSTRATION BY SUMAN MAHARAJAN, KATHMANDU, NEPAL; CC LICENSE UNDER PREVENTION OF CORNEAL BLINDNESS COMMUNITY AWARENESS POSTER-NEPAL © 2023, SAGARMATHA CHOUDHARY EYE HOSPITAL AND LONDON SCHOOL OF HYGIENE AND TROPICAL MEDICINE CC BY-NC-SA 4.0

## Dukhiya: a sad outcome

*If you get injured and the eye gets red,*

*Ignoring it makes it worse instead,*

*Staying at home, hoping it will go,*

*Leads to problems, don't you know?*

**Figure:**